# Occupational Stress and Altered Serum Leptin Among Female Workers in Southern Brazil

**DOI:** 10.3390/diseases14020063

**Published:** 2026-02-09

**Authors:** Cláudia Lorenzi Caberlon, Janaína Cristina da Silva, Anderson Garcez, Harrison Canabarro de Arruda, Ingrid Stähler Kohl, Heloísa Marquardt Leite, Maria Teresa Anselmo Olinto, Heloísa Theodoro

**Affiliations:** 1 Post-Graduate Program in Health Sciences, University of Caxias do Sul, UCS, Caxias do Sul 95070-560, RS, Brazil; claudiacaberlon@hotmail.com; 2 Post-Graduate Program in Collective Health, University of Vale do Rio dos Sinos, Unisinos, São Leopoldo 93022-750, RS, Brazil; jcsilvanutri2@gmail.com; 3 Post-Graduate Program in Medical Sciences: Endocrinology, Federal University of Rio Grande do Sul, UFRGS, Porto Alegre 90610-264, RS, Brazil; adsgarcez@gmail.com (A.G.); ingridkohl.nutri@gmail.com (I.S.K.); 4 Post-Graduate Program in Food, Nutrition and Health, Federal University of Rio Grande do Sul, UFRGS, Porto Alegre 90610-264, RS, Brazil; harrisoncanabarro@gmail.com; 5 Faculty of Medicine, Federal University of the Southern Border, UFFS, Chapecó 89815-899, SC, Brazil; heloo_m@hotmail.com

**Keywords:** leptin, occupational stress, shift work, women

## Abstract

Background: Leptin is a peptide hormone produced by white adipose tissue and is essential for the regulation of appetite and energy homeostasis. Evidence suggests that leptin concentrations may vary according to emotional and psychosocial conditions, such as occupational stress, particularly in vulnerable populations, including female workers. Accordingly, this study aimed to examine the association between occupational stress and altered serum leptin levels among female workers. Methods: A cross-sectional design was applied to a sample of 302 female workers employed in plastic manufacturing plants in southern Brazil. Serum leptin concentrations were quantified, and values exceeding 15.2 ng/mL were considered elevated. Occupational stress was measured using the short version of the Job Stress Scale. Associations between occupational stress and elevated leptin levels were estimated using Poisson regression models with robust variance adjustment. Results: The mean age of the participants was 35.4 years (SD [standard deviation] = 10.1). Elevated serum leptin levels were observed in 78.1% of the sample (95% CI: 73.5–82.8), whereas 21.9% (95% CI: 17.2–26.5) were classified as experiencing occupational stress. After controlling for relevant confounding variables, including obesity, women experiencing occupational stress showed a 15% higher probability of elevated leptin levels compared with those without occupational stress (prevalence ratio [PR] = 1.15; 95% CI: 1.02–1.30; *p* = 0.022). Conclusions: Occupational stress was independently linked to increased serum leptin concentrations among female workers, suggesting that psychosocial stressors in the workplace may play a role in the disruption of leptin regulation in this population.

## 1. Introduction

Leptin is a peptide hormone synthesized by white adipose tissue and plays a central role in the regulation of appetite and energy metabolism [1]. The primary site of leptin action is the brain, particularly the brainstem and the hypothalamus, where it modulates satiety and suppresses food intake. Elevated leptin levels signal to the nervous system the need to reduce energy consumption, whereas low leptin concentrations stimulate increased food intake to replenish energy stores [2,3]. In individuals with obesity, despite the presence of hyperleptinemia—defined as elevated circulating leptin levels—a diminished physiological response to its anorexigenic effects is observed, a phenomenon known as leptin resistance [4,5]. This condition is associated with multiple mechanisms that are not yet fully elucidated, among which chronic low-grade inflammation characteristic of obesity is particularly prominent, especially hypothalamic inflammation mediated by proinflammatory cytokines [6,7]. Additionally, previous findings suggest that leptin may play a role in the development of obesity in response to stress, particularly among women [8].

According to the International Labour Organization (ILO), occupational stress refers to an adverse emotional state that arises when job-related demands exceed a worker’s capacity to cope, given the resources, competencies, and skills available to them [9]. Although hormones such as cortisol, epinephrine, pro-inflammatory cytokines, and melatonin have traditionally been used as biomarkers of stress, emerging—albeit still limited—evidence suggests that ghrelin and leptin are also modulated by stress and may serve as potential biomarkers of this condition [10,11,12,13]. In addition, women may be particularly vulnerable to occupational stress due to the cumulative burden of dual roles, encompassing paid employment alongside domestic and caregiving responsibilities [14].

Studies examining the relationship between leptin and stress remain limited. A pioneering study conducted in Taiwan reported significantly elevated serum leptin levels among individuals with persistent posttraumatic stress symptoms, even after adjustment for age, sex, and body mass index, suggesting a potential involvement of leptin in hypothalamic–pituitary–adrenal (HPA) axis-related stress responses [15]. These findings support the notion that leptin, beyond its classical role in appetite regulation, may be associated with chronic psychological stress and stress-related metabolic dysfunction. In contrast, a study involving 161 healthcare workers found an inverse association between occupational stress and leptin levels, whereby reduced job control—particularly following night shifts—was associated with lower circulating leptin concentrations [16]. The authors proposed that leptin may serve as a biomarker of occupational stress, especially in work environments characterized by high demands, limited decision latitude, and night work.

Among women workers, several occupational factors have been explored in relation to health outcomes, including shift work and other nonstandard work schedules. Shiftwork, characterized by irregular schedules that deviate from the conventional daytime pattern, has become increasingly prevalent, largely driven by the demand for continuous production across multiple sectors, particularly within the industrial workforce [17]. Individuals engaged in shift-based work, particularly those working night shifts, are at an increased risk of adverse health outcomes [18], notably obesity and occupational stress [19,20,21,22,23]. These effects are largely attributed to the disruption of circadian rhythms associated with nonstandard work schedules [24,25].

Although leptin has been extensively studied in the context of metabolic regulation, its relationship with occupational stress remains insufficiently understood, particularly in working populations. Existing studies addressing leptin and stress-related processes have reported inconsistent findings, and evidence focusing on women in occupational settings is still limited. Female workers may be especially vulnerable to stress-related physiological alterations due to the interaction between occupational demands, work schedules, and biological factors. Within this context, the present study aimed to investigate the association between occupational stress and altered serum leptin levels among female workers. By addressing this gap, the study contributes to a better understanding of the interplay between occupational stress, work-related factors, and metabolic biomarkers in women, with potential implications for occupational health and disease prevention. We hypothesized that higher levels of occupational stress would be associated with elevated serum leptin concentrations.

## 2. Materials and Methods

### 2.1. Study Design and Population

This cross-sectional investigation was carried out with female employees working at an industrial site dedicated to the production of plastic goods and household products, situated in the metropolitan region of Porto Alegre, Rio Grande do Sul, Brazil. The present analysis forms part of a broader research initiative entitled Health Conditions of Female Shift Workers: A Longitudinal Study on Occupational Health (ELO Saúde). Ethical clearance was granted by the Research Ethics Committee of the University of Vale do Rio dos Sinos (CAAE: 53762521.7.0000.5344; protocol code: 5681627; date of approval: 7 March 2022). Prior to enrollment, all participants provided written informed consent. The study procedures complied with established ethical guidelines for research involving human subjects, ensuring participant confidentiality and anonymity, and were conducted in accordance with the Declaration of Helsinki.

### 2.2. Sample and Sampling

The study population comprised all female employees eligible during the study period. Eligibility criteria included women aged 18 years or older who were employed in either production-line or administrative positions at the company. Women were excluded if they were pregnant, on temporary leave from work activities, or had been employed for less than three months. A total of 546 women met the eligibility criteria and were invited to participate in the study. Of these, 452 completed the interview stage, accounting for refusals and follow-up losses. All interviewed participants were subsequently invited to provide venous blood samples for biochemical analyses, including serum leptin determination. Complete laboratory data were obtained from 302 participants, including 232 production workers and 70 administrative employees. All participants worked 44 h per week. Production workers followed a six-day work schedule with one day off and were assigned to one of three fixed shifts (morning, afternoon, or night), whereas administrative employees worked regular daytime administrative hours on a five-day schedule with two days off. The final analytical sample provided 80% statistical power to identify prevalence ratio (PR) effect sizes of 0.14 or higher, assuming a 95% confidence interval (CI).

### 2.3. Data Collection and Instruments

Data collection was conducted between August 2022 and March 2023. Access to the company was formally authorized by the company’s executive management and the occupational health department of the corporate group. Information was obtained using a structured, pre-coded, and pre-tested questionnaire administered through face-to-face interviews at the workplace by trained interviewers following standardized procedures. Anthropometric measurements were performed immediately after the interviews. A pilot study was conducted prior to data collection to test the instruments and ensure consistency across interviewers. Venous blood samples were collected after completion of the interviews, either on the same day or on subsequent days, depending on participant availability. Biological specimens were obtained by qualified personnel during the morning hours (7:00–10:00 a.m.), either in a designated area within the workplace or at participants’ homes. Participants were instructed to fast for 8–12 h, abstain from alcohol for 72 h, avoid caffeine intake, and refrain from intense physical activity for at least 24 h prior to sample collection. To ensure data quality, approximately 10% of the interviews were re-evaluated by telephone using a shortened questionnaire containing items with expected response stability. Biochemical analyses were performed by a certified external clinical laboratory. All collected data were subsequently coded and reviewed by the study supervisors.

### 2.4. Outcome: Serum Leptin

Serum leptin concentrations were measured using serum samples with a minimum required volume of 0.5 mL. Specimens were maintained under frozen storage conditions and considered stable for up to 30 days at temperatures between −5 °C and −25 °C, with −20 °C defined as the optimal storage temperature. Quantification was performed using an enzyme-linked immunosorbent assay (ELISA), and results were expressed as nanograms per milliliter (ng/mL). Serum leptin was measured at a certified external clinical laboratory using standardized and validated immunoassay procedures, in accordance with the manufacturer’s instructions. In brief, serum specimens, along with calibration standards and quality control samples, were dispensed into microplate wells pre-coated with anti-leptin antibodies. After successive washing procedures, an enzyme-conjugated secondary antibody was applied and incubated according to the manufacturer’s instructions. A chromogenic substrate was then added, and absorbance was measured using a microplate reader. Leptin concentrations were derived from a standard calibration curve. Serum leptin levels ranging from 0.5 to 15.2 ng/mL were considered within the reference interval, while values exceeding 15.2 ng/mL were classified as elevated [26]. Although a universal cutoff for elevated leptin has not been established, leptin concentrations above approximately 15 ng/mL have been used in previous studies involving adult women [27,28].

### 2.5. Main Exposure: Occupational Stress

Occupational stress was assessed using the short version of the Job Stress Scale (JSS), originally developed by Karasek (1979) [29] and later adapted and validated for use in Brazilian Portuguese by Alves et al. (2004) [30]. The instrument comprises 17 items rated on a four-point Likert scale ranging from 1 (never) to 4 (often) and evaluates four dimensions: psychological demand (5 items), intellectual discretion (6 items), decision authority (2 items), and social support (4 items) [29,30]. Job control was calculated by summing the scores of the intellectual discretion and decision authority subscales, as proposed in the original demand–control model. Psychological demand and job control scores were dichotomized into high and low categories based on the sample-specific median, in accordance with recommended procedures for the Job Stress Scale [29,30]. Occupational stress (job strain) was defined as the concurrent presence of high psychological demand and low job control. In the present study, internal consistency was moderate, with Cronbach’s alpha coefficients of 0.58 for the psychological demand subscale and 0.54 for the job control subscale.

### 2.6. Covariates

To describe the characteristics of the study population and account for potential confounders in multivariable analyses, data on demographic, socioeconomic, occupational, behavioral, and health-related factors were comprehensively obtained.

Demographic and socioeconomic variables included age, self-reported skin color, marital status, educational level, and household socioeconomic status. Age was recorded in completed years at the time of the interview and grouped into three categories (18–30, 31–40, and ≥41 years). Self-reported skin color was classified as White or Other, the latter including Black, Brown, Indigenous, and Yellow (Asian). Marital status was categorized as living without a partner (single, separated, divorced, or widowed) or living with a partner (married or cohabiting). Educational level was defined according to total years of schooling completed and grouped as primary education (≤8 years), secondary education (9–11 years), or technical/higher education (≥12 years). Household socioeconomic status was assessed using per capita family income, calculated as the total household income reported for the previous month divided by the number of residents in the household and categorized relative to the Brazilian national minimum wage (<1, 1–2, or >2 minimum wages; minimum wage in 2022 = BRL 1212.00).

Occupational characteristics focused on work schedule. In the production sector, employees were assigned to one of three fixed shifts: morning (6:00 a.m. to 2:00 p.m.), afternoon (2:00 p.m. to 10:00 p.m.), or night (10:00 p.m. to 6:00 a.m.). Administrative personnel worked exclusively during daytime hours, between 7:00 a.m. and 7:00 p.m. Using recorded clock-in and clock-out data, participants were subsequently categorized as day-shift workers (working between 6:00 a.m. and 10:00 p.m.) or night shift workers (working between 10:00 p.m. and 6:00 a.m.).

Behavioral and health-related variables included sleep quality, physical activity, smoking status, alcohol consumption, self-perceived health, symptoms of common mental disorders, dietary habits, and nutritional status. Subjective sleep quality was assessed by the question “During the last month, how would you rate your sleep overall?” and dichotomized as very good/good or poor/very poor. Engagement in leisure time physical activity was determined by self-reported participation in sports, exercise, or recreational physical activities during the previous week, excluding commuting, and classified as yes or no. Smoking status was categorized as never smoker or current/former smoker. Alcohol consumption was defined as no consumption (none or less than once per week) or yes (at least once per week during the previous year). Self-perceived health status was measured using a five-point Likert scale and grouped into excellent/very good, good, or fair/poor.

Symptoms of common mental disorders were evaluated using the validated 20-item Self-Reporting Questionnaire (SRQ-20), a yes/no screening instrument that captures psychological and somatic symptoms experienced in the previous 30 days, with scores of eight or more positive responses indicating the presence of such symptoms [31]. Participants also reported the number of meals typically consumed per day, which was categorized as ≤3 or ≥4 meals.

Nutritional status was assessed based on body mass index (BMI), defined as body weight in kilograms divided by height in meters squared. Body weight and stature were measured using a calibrated digital scale (Omron^®^, model HN-289; capacity 150 kg; accuracy 0.1 kg; OMRON Healthcare, São Paulo, Brazil) and a portable stadiometer (Balmak^®^; maximum height 2.1 m; accuracy 1 mm; BALMAK, São Paulo, Brazil), respectively. Measurements were performed in duplicate under standardized conditions, with participants barefoot, wearing light clothing, standing upright, and with arms relaxed alongside the body. According to BMI thresholds, participants were categorized as normal weight (BMI < 25 kg/m^2^), overweight (25 ≤ BMI < 30 kg/m^2^), or obesity (BMI ≥ 30 kg/m^2^) [32].

### 2.7. Statistical Analyses

Data processing and management were conducted using EpiData software (version 3.1; Centers for Disease Control and Prevention, Atlanta, GA, USA). Data were entered independently by two operators, and the resulting datasets were subjected to systematic consistency checks to identify and correct potential entry errors, thereby ensuring data quality. Descriptive statistical analyses were performed to summarize the characteristics of the study sample and to examine the distribution of elevated serum leptin concentrations. Continuous variables were presented as means and standard deviations, whereas categorical variables were expressed as absolute frequencies and proportions. Differences in the distribution of occupational stress and the prevalence of altered serum leptin across categories of sample characteristics were evaluated using Fisher’s exact test for comparison of proportions.

Associations between occupational stress and altered serum leptin were investigated by estimating prevalence ratios (PRs) and 95% confidence intervals (95% CIs) through Poisson regression models with robust variance estimation [33]. Variables with a *p*-value ≤ 0.20 in bivariate analyses with either the exposure or the outcome were selected for inclusion in the multivariable analysis. A hierarchical analytical strategy was adopted for multivariable adjustment [34], including three sequential models: Model I consisted of the crude (unadjusted) analysis; Model II included adjustment for demographic and socioeconomic factors; and Model III further incorporated occupational characteristics and behavioral and health-related variables.

All statistical analyses were performed using Stata (version 14.0; StataCorp LP, College Station, TX, USA). A two-tailed *p*-value of less than 0.05 was considered indicative of statistical significance.

## 3. Results

The final analytical sample comprised 302 female employees between 18 and 64 years of age, with a mean age of 35.4 ± 10.1 years. The overall profile of the study population is presented in Table 1. Approximately one third of the participants (35.1%) were aged 18–30 years, 69.5% self-reported white skin color, and just over half (52.0%) reported not living with a partner. Slightly more than half of the women (54.0%) had completed secondary education, and 42.1% reported a per capita household income equivalent to 1–2 minimum wages. With respect to work schedules, 13.2% of the sample was engaged in night shift work. Regarding behavioral and health-related characteristics, 31.7% reported poor or very poor sleep quality, 70.5% indicated no participation in leisure time physical activity, 23.8% were current or former smokers, and 31.1% reported alcohol consumption at least once per week over the previous year. In addition, 28.8% of participants rated their health status as fair or poor and 46.7% had common mental disorders symptoms. Concerning eating patterns, 61.9% reported consuming four or more meals per day. Based on body mass index classification, most participants were categorized as overweight (37.4%) or obesity (30.1%).

The estimated mean serum leptin level among participants was 33.6 ng/mL (95% CI: 30.6–36.6). The prevalence of elevated serum leptin in the study population was 78.1% (95% CI: 73.5–82.8), while 21.9% of participants (95% CI: 17.2–26.5) were classified as experiencing occupational stress. As detailed in Table 1, a significantly higher prevalence of elevated serum leptin was observed among night shift workers, women who did not report regular physical activity, and those with obesity. Regarding occupational stress, a higher prevalence was observed among participants with a per capita household income of one to two minimum wages and among those presenting symptoms of common mental disorders.

Table 2 summarizes the results of the analysis examining the relationship between occupational stress and serum leptin alterations. In this sample of Brazilian female workers, exposure to occupational stress was significantly associated with elevated leptin concentrations. In fully adjusted models, including obesity as a covariate, women classified as experiencing occupational stress had a 15% higher probability of elevated serum leptin compared with those not exposed to occupational stress (PR = 1.15; 95% CI: 1.02–1.30; *p* = 0.022).

## 4. Discussion

In the present investigation, a substantial proportion of female workers employed at an industrial complex in southern Brazil exhibited elevated serum leptin concentrations (>15.2 ng/mL). In addition, multivariable-adjusted analyses indicated that participants experiencing occupational stress had a 15% higher probability of presenting elevated leptin levels compared with their counterparts without occupational stress.

Research examining the association between stress and leptin remains relatively scarce. Among the first investigations to specifically address the link between psychological stress and circulating leptin concentrations was a study conducted in Taiwan by Liao and colleagues [15]. The authors evaluated individuals with persistent posttraumatic stress disorder symptoms after an 18-month follow-up period and reported significantly higher leptin levels in this group compared with controls, even after adjustment for body mass index [15]. Although the type of stress evaluated differs (posttraumatic versus occupational), these findings support the hypothesis that chronic psychological stress, regardless of its source, may influence leptin levels through shared biological mechanisms. Specifically, the physiological pathways affecting leptin regulation appear to be commonly activated across different forms of stress. However, the available literature reports conflicting findings. A previous systematic review and meta-analysis of seven studies demonstrated an overall reduction in circulating leptin concentrations following acute stress exposure, indicating that leptin responses vary according to sex and adiposity, with more pronounced changes observed among normal-weight individuals [8]. These findings suggest that leptin is a dynamic hormone sensitive to stress-related stimuli and may contribute to the mechanistic link between stress, appetite regulation, and metabolic dysregulation.

Also, another study involving 161 healthcare professionals examined the relationship between occupational stress, shift work, and leptin levels and identified an opposing pattern [16]. In that study, low job control—particularly following night shifts—was associated with reduced serum leptin concentrations, suggesting that limited occupational autonomy may lead to stress-mediated hormonal dysregulation [16]. According to the authors, leptin may function as a biomarker of occupational stress, especially in settings characterized by high job demands, low decision latitude, and night work. Notably, the study did not adjust this association for participants’ nutritional status. Accounting for nutritional status is essential, as adiposity strongly influences leptin concentrations and may modulate the physiological response to stress. Leptin is a hormone primarily secreted by adipose tissue, with circulating levels increasing proportionally to fat mass accumulation [35], which may reflect a state of leptin resistance in individuals with obesity [36].

These contradictory findings—namely, increased leptin levels in individuals exposed to posttraumatic stress versus reduced leptin levels in those experiencing occupational stress—underscore the need for further studies investigating the physiological mechanisms underlying stress-related leptin regulation. Nevertheless, the association between occupational stress and altered leptin levels observed in our study, which remained significant after adjustment for nutritional status, supports this hypothesis and highlights chronic stress as a relevant contributor to hormonal dysregulation. Chronic stress is widely recognized as one of the major drivers of physiological dysfunction in contemporary societies, exerting systemic effects that directly contribute to the development and persistence of metabolic disorders, such as obesity. Several physiological pathways link chronic stress to metabolic alterations, including disruption of circadian rhythms and dysregulation of the neuroendocrine system [37]. The physiological stress response is initially adaptive; however, when stress becomes chronic, it leads to sustained activation of the hypothalamic–pituitary–adrenal (HPA) axis. Hyperactivation of this axis results in increased cortisol secretion, a hormone that plays a central role in the regulation of metabolism, sleep, and appetite. Excess cortisol promotes visceral fat accumulation, increases insulin resistance, and enhances food intake, particularly of energy-dense foods rich in sugars and fats. Consequently, this physiological state may create a favorable environment for the development of obesity [37].

Although leptin is a hormone whose circulating concentration generally reflects the amount of body fat, chronic stress may increase leptin levels independently of nutritional status. Under conditions of chronic stress, elevated cortisol stimulates leptin production by adipocytes. In addition, stress may induce leptin resistance, leading the organism to increase leptin secretion as a compensatory mechanism. Consequently, even eutrophic individuals may exhibit elevated leptin levels when exposed to chronic stress, which may contribute to the development of metabolic alterations and obesity. In contrast, an animal model study demonstrated that repeated exposure to acute stress results in a transient reduction in appetite and body weight, without significantly altering serum leptin levels [38]. In the present study, the significant association between occupational stress and altered leptin levels, even among eutrophic women, reinforces the hypothesis that chronic stress may promote hormonal changes independent of nutritional status. In this context, beyond the transient effects of acute stress, chronic occupational stress may be associated with persistent alterations in leptin levels, regardless of nutritional status.

Another important physiological aspect concerns the impact of chronic stress on circadian rhythms, particularly among individuals exposed to night work or irregular shift schedules [39]. Shift workers experience circadian misalignment due to the inversion of sleep–wake cycles, which constitutes a chronic stressor. Disruption of the sleep–wake cycle directly affects the secretion of melatonin, cortisol, insulin, and appetite-regulating hormones, thereby contributing to increased adiposity. Sleep deprivation or poor sleep quality—common among shift workers—as well as altered meal timing have been identified as additional risk factors, resulting in significant metabolic disturbances [40,41,42]. Stress manifests not only as a psychological response but also as a physiological factor. Beyond the aforementioned effects, chronic stress negatively influences food intake through alterations in appetite-regulating hormones, such as leptin and ghrelin [37]. Moreover, stress not only activates the hypothalamic–pituitary–adrenal (HPA) axis but may also impair endogenous defense mechanisms that help neutralize toxic substances produced in excess during stress, known as free radicals. When these radicals accumulate, they can induce cellular damage, promote inflammation, and contribute to the development of chronic diseases, including obesity [43].

**Figure 1:**
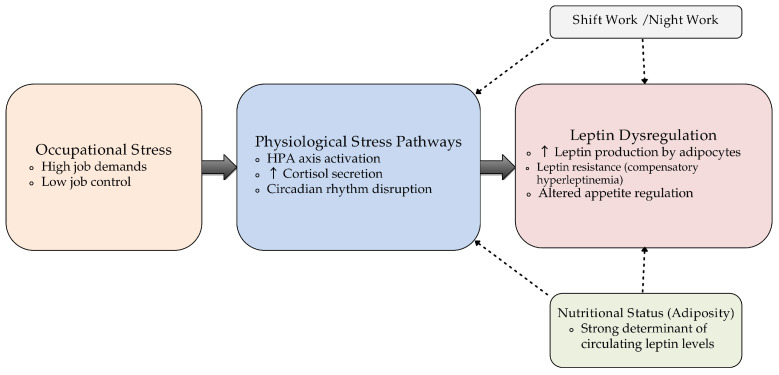
Conceptual model illustrating the proposed relationship between occupational stress and leptin dysregulation.

Figure 1 presents a conceptual model illustrating the proposed relationships between occupational stress and leptin dysregulation.

This study demonstrates several relevant strengths from both methodological and conceptual perspectives. To the best of our knowledge, relatively few investigations have examined the association between occupational stress and circulating leptin levels in a clearly defined population of Brazilian female workers, including women engaged in shift-based work. By focusing on this specific occupational context, the present analysis addresses a workforce that may be at elevated risk for metabolic and endocrine disturbances, thereby contributing to an underexplored area of the literature. The findings extend current evidence by providing additional insight into the potential biological pathways through which occupational stress may influence leptin regulation and metabolic processes, particularly among women exposed to nonstandard work schedules. The robustness of the study design was reinforced by the adoption of standardized data collection procedures and the involvement of trained field staff, thereby minimizing the potential for systematic measurement bias. In addition, the use of objectively measured serum leptin levels, together with the adjustment for sociodemographic, behavioral, and health-related variables, enabled a more thorough control of potential confounders.

Collectively, these methodological aspects contribute to the internal coherence and reliability of the study results. Nonetheless, certain limitations warrant consideration. Owing to the cross-sectional nature of the study, in which exposure and outcome were assessed concurrently, causal relationships cannot be established, and the possibility of reverse or bidirectional associations cannot be excluded. Furthermore, the sample was composed exclusively of female workers from a single industrial site in southern Brazil, which may restrict the generalizability of the findings to other occupational contexts, geographic locations, or to male populations. The exclusive focus on fixed-shift schedules precluded the evaluation of potential effects related to rotating or irregular shift patterns. Notably, the subgroup of women classified as night workers consisted solely of individuals assigned to fixed night shifts. At the same time, while work shift was explored as a potential confounding factor in the association between occupational stress and altered leptin levels, the relatively small sample size of this subgroup limited the statistical power to detect potential effects of night work on hormone secretion. Therefore, findings related to night work should be interpreted with caution, and future studies with larger samples and diverse night work schedules are warranted. In addition, although sensitivity analyses indicated that the inclusion of administrative employees did not meaningfully affect the main findings, the relatively small size of this subgroup limited the feasibility of more comprehensive adjusted or stratified analyses. Accordingly, this should be regarded as a potential limitation of the study, particularly considering possible differences in occupational stress exposure between administrative and production roles. Another important limitation relates to the assessment of occupational stress, which was based on the short version of the Job Stress Scale, a culturally adapted and validated instrument for use in Brazilian Portuguese. Although appropriate for epidemiological research, this measure may not fully capture clinically diagnosed stress conditions, thereby introducing the possibility of exposure misclassification. The Job Stress Scale also does not distinguish between acute and chronic stress exposure, which may differentially influence serum leptin levels. Furthermore, several relevant biological and clinical variables—such as circulating glucocorticoid levels, menstrual cycle phase, and the use of sleep-related pharmacological treatments (e.g., for obstructive sleep apnea)—were not assessed and may have influenced circulating leptin concentrations. Also, several behavioral, biological, and occupational-related factors that may modulate occupational stress—such as lifestyle habits, individual coping strategies, comorbid conditions, and psychosocial characteristics—were not specifically evaluated and should be considered in future studies to better elucidate their influence on stress-related responses and leptin regulation. Finally, serum leptin was measured at a single time point due to logistical and operational constraints inherent to data collection in an occupational setting. Although information on work shift at the time of blood collection was available, the timing of sampling in relation to night work could not be systematically standardized or analytically adjusted, and no correction for circadian rhythmicity was performed. Dynamic leptin assessment could enhance the robustness of the findings, particularly considering the well-established circadian variation in leptin secretion, characterized by lower daytime levels and higher nocturnal concentrations; therefore, this methodological limitation should be considered when interpreting single–time-point hormonal measurements.

In light of these considerations, future research should prioritize longitudinal study designs, include participants from multiple companies and occupational sectors, and incorporate additional biological and circadian factors. Such approaches will be essential for more clearly elucidating the relationship between occupational stress and leptin dysregulation among women workers.

## 5. Conclusions

In this population of female workers, occupational stress was independently associated with elevated serum leptin concentrations, even after adjustment for obesity and other relevant confounding factors. These findings indicate that psychosocial stressors in the workplace may contribute to altered leptin regulation. The results further highlight the increased metabolic vulnerability of women workers, particularly those exposed to occupational stress. From a public health and occupational health perspective, these findings support the implementation of targeted workplace interventions aimed at reducing psychosocial stress and mitigating metabolic risk among female workers.

## Figures and Tables

**Table 1:** Sample characteristics and prevalence of occupational stress and altered serum leptin (>15.2 ng/mL) according to sample characteristics among Brazilian female workers, 2022. (n = 302).

Characteristics	n (%)	Occupational Stress		Prevalence of Altered Leptin	
		n (%)	*p*-Value *	n (%)	*p*-Value *
***Age***			0.270		0.661
18–30 years	106 (35.1)	26 (24.5)		83 (78.3)	
31–40 years	103 (34.1)	17 (16.5)		83 (80.6)	
≥41 years	93 (30.8)	23 (24.7)		70 (75.3)	
***Skin color***			0.096		0.880
White	210 (69.5)	40 (19.1)		163 (77.6)	
Other	92 (30.5)	26 (28.3)		73 (79.3)	
***Marital status***			0.578		0.677
Without partner	157 (52.0)	32 (20.4)		121 (77.1)	
With partner	145 (48.0)	34 (23.5)		115 (79.3)	
***Educational level***			0.200		0.705
Primary school	21 (7.0)	4 (19.1)		18 (85.7)	
Secondary school	163 (54.0)	42 (25.8)		125 (76.7)	
Technical/higher	118 (39.1)	20 (17.0)		93 (78.8)	
***Per capita family income***			0.001		0.654
<1 minimum wage	111 (36.8)	27 (24.3)		86 (77.5)	
1–2 minimum wages	127 (42.1)	35 (27.6)		102 (80.3)	
>2 minimum wages	63 (20.9)	4 (6.4)		47 (74.6)	
***Work shift***			0.681		0.047
Day shift	262 (86.8)	56 (21.4)		201 (76.7)	
Night shift	40 (13.2)	10 (25.0)		35 (87.5)	
***Sleep quality***			0.767		0.881
Very good/good	206 (68.2)	44 (21.4)		160 (77.7)	
Poor/very poor	96 (31.7)	22 (22.9)		76 (79.2)	
***Physical activity***			0.126		0.002
Yes	89 (29.5)	14 (15.7)		59 (63.3)	
No	213 (70.5)	52 (24.4)		177 (83.1)	
***Current smoking status***			0.627		0.627
Never smoked	230 (76.1)	52 (22.6)		178 (77.4)	
Current smoker/former smoker	72 (23.8)	14 (19.4)		58 (80.6)	
***Alcohol consumption***			0.882		0.367
No consumption	208 (68.9)	45 (21.6)		159 (76.4)	
≥1 time a week	94 (31.1)	21 (22.3)		77 (81.9)	
***Self-perception of health***			0.262		0.142
Excellent/Very good	73 (24.2)	11 (15.1)		57 (78.1)	
Good	142 (47.0)	35 (24.7)		105 (73.4)	
Fair/poor	87 (28.8)	20 (23.0)		74 (85.1)	
***Number of meals per day***			0.564		0.569
3 or fewer	115 (38.1)	23 (20.0)		92 (80.0)	
4 or more	187 (61.9)	43 (23.0)		144 (77.0)	
***Common mental disorders symptoms***			0.012		0.070
No (SRQ-20 < 8)	161 (53.3)	26 (16.2)		119 (73.9)	
Yes (SRQ-20 ≥ 8)	141 (46.7)	40 (28.4)		117 (83.0)	
***Nutritional status***			0.165		<0.001
Normal (BMI < 25 kg/m^2^)	98 (32.5)	20 (20.4)		52 (53.1)	
Overweight (25 kg/m^2^ ≤ BMI < 30 kg/m^2^)	113 (37.4)	31 (27.4)		96 (85.0)	
Obesity (BMI ≥ 30 kg/m^2^)	91 (30.1)	15 (16.5)		88 (96.7)	

BMI, Body Mass Index. SRQ-20, Self-Reporting Questionnaire. * Fisher’s exact test for comparison of proportions.

**Table 2:** Unadjusted and adjusted prevalence ratios (PRs) and their respective 95% confidence intervals (95% CIs) for the association between occupational stress and altered serum leptin (>15.2 ng/mL) among Brazilian female workers, 2022. (n = 302).

	Serum Leptin (>15.2 ng/mL)	Model I	Model II	Model III
Occupational stress	n (%)	PR (95% CI)	PR (95% CI)	PR (95% CI)
No	179 (75.9)	1.00	1.00	1.00
Yes	57 (86.4)	1.14 (1.01–1.28)	1.14 (1.01–1.29)	1.15 (1.02–1.30)
*p*-value *		0.034	0.041	0.022

* *p*-value for the Wald test for heterogeneity of proportions obtained by means of Poisson regression with robust variance. Model I: Unadjusted (crude) analysis; Model II: Analysis adjusted for demographic and socioeconomic characteristics (skin color, educational level, and per capita family income); Model III: Analysis adjusted for Model II + occupational characteristic (work shift) and behavioral and health characteristics (leisure time physical activity, self-perception of health, common mental disorders symptoms, and nutritional status [obesity]).

## Data Availability

The dataset will be made available on request from the authors. The raw data supporting the conclusions of this article will be made available by the authors on request.

## Abbreviations

The following abbreviations are used in this manuscript:

ILO	International Labour Organization
HPA	Hypothalamic–Pituitary–Adrenal
BMI	Body Mass Index
PR	Prevalence Ratio
CI	Confidence Interval
EIA	Enzyme Immunoassay
